# Bis(4-acetyl-3-methyl-1-phenyl-1*H*-pyrazol-5-olato-κ^2^ *O*,*O*′)bis­(*N*,*N*-dimethyl­formamide-κ*O*)nickel(II)

**DOI:** 10.1107/S1600536810026231

**Published:** 2010-07-10

**Authors:** Hualing Zhu, Zhen Wei, Luxia Bu, Xiaoping Xu, Jun Shi

**Affiliations:** a Department of Basic Science, Tianjin Agriculturial College, Tianjin Jinjing Road No 22, Tianjin 300384, People’s Republic of China

## Abstract

The title complex, [Ni(C_12_H_11_N_2_O_2_)_2_(C_3_H_7_NO)_2_], lies on on an inversion center. The Ni^II^ ion is coordinated in a slightly distorted octa­hedral coordination enviroment by four O atoms from two bis-chelating 4-acety-3-methyl-1-phenyl-1*H*-pyrazol-5-olate ligands in the equatorial plane and two O atoms from two *N*,*N*-dimethyl­formamide ligands in the axial sites. In the crystal structure, weak inter­molecular π–π stacking inter­actions with centroid–centroid distances of 3.7467 (13) Å link mol­ecules into chains extending alongthe *b* axis.

## Related literature

For related structures: Shi *et al.* (2005 ▶); Zhu *et al.* (2010*a* ▶,*b* ▶, 2005 ▶).
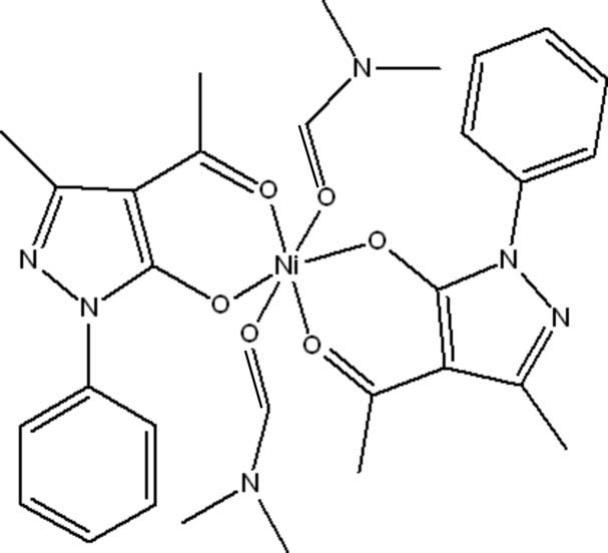


## Experimental

### 

#### Crystal data

[Ni(C_12_H_11_N_2_O_2_)_2_(C_3_H_7_NO)_2_]
*M* *_r_* = 635.36
Monoclinic, 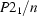

*a* = 8.7201 (17) Å
*b* = 17.119 (3) Å
*c* = 9.852 (2) Å
β = 101.56 (3)°
*V* = 1440.9 (5) Å^3^
*Z* = 2
Mo *K*α radiation
μ = 0.73 mm^−1^
*T* = 113 K
0.20 × 0.18 × 0.10 mm

#### Data collection

Rigaku Saturn CCD diffractometer
Absorption correction: multi-scan (*CrystalClear*; Rigaku, 2008 ▶) *T*_min_ = 0.868, *T*_max_ = 0.931
10320 measured reflections
2529 independent reflections
2279 reflections with *I* > 2σ(*I*)
*R*_int_ = 0.031

#### Refinement

*R*[*F* ^2^ > 2σ(*F* ^2^)] = 0.030
*wR*(*F* ^2^) = 0.078
*S* = 1.09
2529 reflections
200 parameters
H-atom parameters constrained
Δρ_max_ = 0.32 e Å^−3^
Δρ_min_ = −0.58 e Å^−3^

### 

Data collection: *CrystalClear* (Rigaku, 2008 ▶); cell refinement: *CrystalClear*; data reduction: *CrystalClear*; program(s) used to solve structure: *SHELXS97* (Sheldrick, 2008 ▶); program(s) used to refine structure: *SHELXL97* (Sheldrick, 2008 ▶); molecular graphics: *SHELXTL* (Sheldrick, 2008 ▶); software used to prepare material for publication: *SHELXTL*.

## Supplementary Material

Crystal structure: contains datablocks I, global. DOI: 10.1107/S1600536810026231/lh5075sup1.cif

Structure factors: contains datablocks I. DOI: 10.1107/S1600536810026231/lh5075Isup2.hkl

Additional supplementary materials:  crystallographic information; 3D view; checkCIF report

## Figures and Tables

**Fig. 1.**
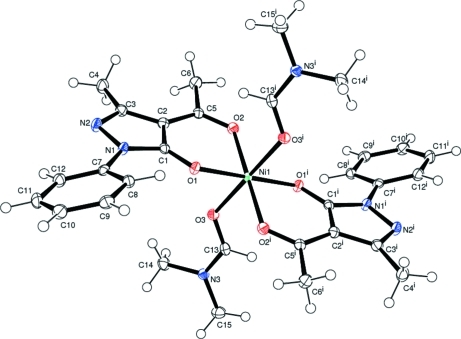
The molecular structure of the title compound with the atom numbering scheme. Displacement ellipsoids are drawn at the 30% probability level. H atoms are presented as a small spheres of arbitrary radius (symmetry code: (i) -x+1, -y+2, -z+1).

**Fig. 2.**
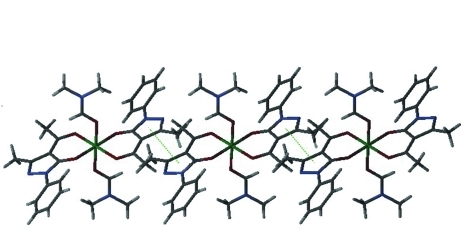
Part of the crystal structure showing intermolecular π–π interactions as dashed lines.

## supplementary crystallographic information

### Comment

As part of our onging studies of pyrazolone derivatives as potential ligands (Zhu *et al.*, 2005; 2010a,b) we report the structure of the title complex, (I).

The molecular structure of the title complex is shown in Fig. 1. The Ni^II^ ion lies on a crystallographic inversion centre and adopts a slightly distorted octahedral coordination environment provided by four O atoms from two 4-acety-3-methyl-1-phenyl-1*H*-pyrazol-5(4*H*)- onato ligands in the equatorial plane two O atoms from two *N*,*N*-bimethylformamide ligands in the axial sites. A related complex has previously been published (Shi *et al.*, 2005). In the crystal structure, weak intermolecular π–π stacking interactions involving the pyrazole rings, with centroid to centroid distances of 3.7467 (13)Å link molecules into one-dimensional chains (Fig 2).

### Experimental

The title compound was synthesized by dropping a nickel acetate (5*m* mol) ethanolic solution into an ethanolic solution of 4-acetyl-3-methyl-1-phenyl-1H-pyrazolone-5 (10*m* mol) and stirring for about 2 h under room temperature. The green blocks which were obtained were dried in air. The product was recrystallized from *N*,*N*-dimethylformamide which afforded crystals suitable for *X*–ray analysis.

### Refinement

All H atoms were geometrically positioned and refined using a riding model, with C—H = 0.95 Å or 0.98 Å for the methyl H atoms and *U*_iso_(H)= 1.2 *U*_eq_(C) or 1.5*U*_eq_(C) for methyl H atoms.

### Crystal data

**Table:**

[Ni(C_12_H_11_N_2_O_2_)_2_(C_3_H_7_NO)_2_]	*F*(000) = 668
*M**_r_* = 635.36	*D*_x_ = 1.464 Mg m^−^^3^
Monoclinic, *P*2_1_/*n*	Mo *K*α radiation, λ = 0.71073 Å
Hall symbol: -P 2yn	Cell parameters from 4482 reflections
*a* = 8.7201 (17) Å	θ = 2.4–27.9°
*b* = 17.119 (3) Å	µ = 0.73 mm^−^^1^
*c* = 9.852 (2) Å	*T* = 113 K
β = 101.56 (3)°	Block, green
*V* = 1440.9 (5) Å^3^	0.20 × 0.18 × 0.10 mm
*Z* = 2	

### Data collection

**Table:**

Rigaku Saturn CCD diffractometer	2529 independent reflections
Radiation source: rotating anode	2279 reflections with *I* > 2σ(*I*)
confocal	*R*_int_ = 0.031
Detector resolution: 7.31 pixels mm^-1^	θ_max_ = 25.0°, θ_min_ = 2.4°
ω and φ scans	*h* = −10→10
Absorption correction: multi-scan (*CrystalClear*; Rigaku, 2008)	*k* = −19→20
*T*_min_ = 0.868, *T*_max_ = 0.931	*l* = −11→11
10320 measured reflections	

### Refinement

**Table:**

Refinement on *F*^2^	Primary atom site location: structure-invariant direct methods
Least-squares matrix: full	Secondary atom site location: difference Fourier map
*R*[*F*^2^ > 2σ(*F*^2^)] = 0.030	Hydrogen site location: inferred from neighbouring sites
*wR*(*F*^2^) = 0.078	H-atom parameters constrained
*S* = 1.09	*w* = 1/[σ^2^(*F*_o_^2^) + (0.048*P*)^2^ + 0.3836*P*] where *P* = (*F*_o_^2^ + 2*F*_c_^2^)/3
2529 reflections	(Δ/σ)_max_ = 0.001
200 parameters	Δρ_max_ = 0.32 e Å^−^^3^
0 restraints	Δρ_min_ = −0.58 e Å^−^^3^

### Special details

**Table:**

Geometry. All e.s.d.'s (except the e.s.d. in the dihedral angle between two l.s. planes) are estimated using the full covariance matrix. The cell e.s.d.'s are taken into account individually in the estimation of e.s.d.'s in distances, angles and torsion angles; correlations between e.s.d.'s in cell parameters are only used when they are defined by crystal symmetry. An approximate (isotropic) treatment of cell e.s.d.'s is used for estimating e.s.d.'s involving l.s. planes.
Refinement. Refinement of *F*^2^ against ALL reflections. The weighted *R*-factor *wR* and goodness of fit *S* are based on *F*^2^, conventional *R*-factors *R* are based on *F*, with *F* set to zero for negative *F*^2^. The threshold expression of *F*^2^ > σ(*F*^2^) is used only for calculating *R*-factors(gt) *etc*. and is not relevant to the choice of reflections for refinement. *R*-factors based on *F*^2^ are statistically about twice as large as those based on *F*, and *R*- factors based on ALL data will be even larger.

### Fractional atomic coordinates and isotropic or equivalent isotropic displacement parameters (Å^2^)

**Table:**

	*x*	*y*	*z*	*U*_iso_*/*U*_eq_	
Ni1	0.5000	1.0000	0.5000	0.01056 (12)	
O1	0.58951 (13)	1.05337 (7)	0.68398 (11)	0.0138 (3)	
O2	0.41750 (13)	0.91066 (7)	0.60044 (12)	0.0145 (3)	
O3	0.71254 (13)	0.94193 (7)	0.51507 (12)	0.0147 (3)	
N1	0.71370 (16)	1.03649 (8)	0.91454 (14)	0.0136 (3)	
N2	0.73040 (17)	0.97910 (9)	1.01841 (15)	0.0167 (3)	
N3	0.92677 (16)	0.91367 (8)	0.42588 (15)	0.0141 (3)	
C1	0.62073 (19)	1.01164 (9)	0.79249 (18)	0.0121 (4)	
C2	0.57338 (19)	0.93439 (10)	0.82039 (18)	0.0134 (4)	
C3	0.64609 (19)	0.91963 (10)	0.96176 (18)	0.0153 (4)	
C4	0.6431 (2)	0.84786 (11)	1.04694 (19)	0.0208 (4)	
H4A	0.7047	0.8567	1.1404	0.031*	
H4B	0.6880	0.8041	1.0040	0.031*	
H4C	0.5348	0.8356	1.0525	0.031*	
C5	0.46612 (18)	0.88950 (10)	0.72430 (17)	0.0124 (4)	
C6	0.4005 (2)	0.81431 (10)	0.76739 (18)	0.0185 (4)	
H6A	0.3372	0.7888	0.6860	0.028*	
H6B	0.3348	0.8254	0.8350	0.028*	
H6C	0.4865	0.7797	0.8092	0.028*	
C7	0.79488 (19)	1.10812 (10)	0.94735 (17)	0.0134 (4)	
C8	0.74278 (19)	1.17723 (10)	0.87719 (17)	0.0145 (4)	
H8	0.6550	1.1766	0.8025	0.017*	
C9	0.8205 (2)	1.24649 (10)	0.91776 (18)	0.0177 (4)	
H9	0.7846	1.2936	0.8709	0.021*	
C10	0.9504 (2)	1.24833 (11)	1.02602 (19)	0.0206 (4)	
H10	1.0017	1.2963	1.0544	0.025*	
C11	1.0036 (2)	1.17911 (11)	1.09167 (18)	0.0212 (4)	
H11	1.0938	1.1795	1.1640	0.025*	
C12	0.9269 (2)	1.10923 (10)	1.05317 (18)	0.0174 (4)	
H12	0.9646	1.0621	1.0990	0.021*	
C13	0.78377 (19)	0.94216 (10)	0.41739 (18)	0.0138 (4)	
H13	0.7326	0.9641	0.3316	0.017*	
C14	1.0109 (2)	0.87896 (11)	0.5542 (2)	0.0208 (4)	
H14A	0.9697	0.8998	0.6323	0.031*	
H14B	1.1225	0.8916	0.5665	0.031*	
H14C	0.9973	0.8221	0.5500	0.031*	
C15	1.0048 (2)	0.91441 (11)	0.30838 (19)	0.0210 (4)	
H15A	0.9379	0.9406	0.2298	0.031*	
H15B	1.0247	0.8606	0.2826	0.031*	
H15C	1.1044	0.9425	0.3337	0.031*	

### Atomic displacement parameters (Å^2^)

**Table:**

	*U*^11^	*U*^22^	*U*^33^	*U*^12^	*U*^13^	*U*^23^
Ni1	0.01054 (17)	0.01073 (18)	0.00993 (18)	0.00035 (11)	0.00088 (12)	−0.00072 (11)
O1	0.0168 (6)	0.0137 (6)	0.0102 (6)	−0.0004 (5)	0.0011 (5)	0.0003 (5)
O2	0.0146 (6)	0.0137 (6)	0.0149 (7)	−0.0005 (4)	0.0023 (5)	−0.0013 (5)
O3	0.0136 (6)	0.0163 (6)	0.0145 (6)	0.0012 (5)	0.0035 (5)	−0.0007 (5)
N1	0.0177 (7)	0.0117 (8)	0.0104 (7)	−0.0010 (6)	0.0008 (6)	0.0010 (6)
N2	0.0196 (8)	0.0168 (8)	0.0123 (8)	−0.0003 (6)	−0.0004 (6)	0.0034 (6)
N3	0.0129 (7)	0.0145 (8)	0.0150 (7)	0.0012 (5)	0.0031 (6)	0.0008 (6)
C1	0.0097 (8)	0.0145 (9)	0.0122 (9)	0.0020 (6)	0.0022 (6)	−0.0020 (7)
C2	0.0131 (8)	0.0122 (9)	0.0149 (9)	0.0014 (6)	0.0028 (7)	0.0009 (7)
C3	0.0145 (8)	0.0154 (9)	0.0158 (9)	0.0002 (7)	0.0028 (7)	0.0003 (7)
C4	0.0226 (9)	0.0202 (10)	0.0177 (9)	−0.0033 (7)	−0.0006 (7)	0.0049 (8)
C5	0.0113 (8)	0.0128 (8)	0.0134 (9)	0.0039 (6)	0.0032 (7)	−0.0016 (7)
C6	0.0221 (9)	0.0156 (9)	0.0173 (9)	−0.0041 (7)	0.0030 (7)	−0.0005 (7)
C7	0.0147 (8)	0.0157 (9)	0.0106 (8)	−0.0015 (7)	0.0048 (7)	−0.0027 (7)
C8	0.0133 (8)	0.0163 (9)	0.0137 (9)	0.0001 (6)	0.0019 (7)	−0.0011 (7)
C9	0.0207 (9)	0.0152 (9)	0.0180 (9)	−0.0005 (7)	0.0060 (7)	0.0020 (7)
C10	0.0246 (9)	0.0190 (10)	0.0182 (10)	−0.0090 (8)	0.0039 (8)	−0.0015 (8)
C11	0.0196 (9)	0.0264 (11)	0.0151 (9)	−0.0068 (7)	−0.0024 (7)	0.0000 (8)
C12	0.0190 (9)	0.0170 (9)	0.0155 (9)	−0.0010 (7)	0.0018 (7)	0.0022 (7)
C13	0.0141 (8)	0.0104 (8)	0.0153 (9)	0.0000 (6)	−0.0010 (7)	−0.0010 (7)
C14	0.0160 (9)	0.0217 (10)	0.0234 (10)	0.0037 (7)	0.0007 (7)	0.0068 (8)
C15	0.0191 (9)	0.0236 (10)	0.0219 (10)	−0.0001 (7)	0.0083 (8)	−0.0008 (8)

### Geometric parameters (Å, °)

**Table:**

Ni1—O2^i^	2.0301 (12)	C5—C6	1.504 (2)
Ni1—O2	2.0301 (12)	C6—H6A	0.9800
Ni1—O1^i^	2.0402 (12)	C6—H6B	0.9800
Ni1—O1	2.0402 (12)	C6—H6C	0.9800
Ni1—O3^i^	2.0820 (12)	C7—C12	1.390 (3)
Ni1—O3	2.0820 (12)	C7—C8	1.399 (2)
O1—C1	1.269 (2)	C8—C9	1.384 (2)
O2—C5	1.262 (2)	C8—H8	0.9500
O3—C13	1.246 (2)	C9—C10	1.392 (3)
N1—C1	1.376 (2)	C9—H9	0.9500
N1—N2	1.405 (2)	C10—C11	1.385 (3)
N1—C7	1.420 (2)	C10—H10	0.9500
N2—C3	1.313 (2)	C11—C12	1.386 (3)
N3—C13	1.326 (2)	C11—H11	0.9500
N3—C14	1.455 (2)	C12—H12	0.9500
N3—C15	1.456 (2)	C13—H13	0.9500
C1—C2	1.428 (2)	C14—H14A	0.9800
C2—C5	1.417 (2)	C14—H14B	0.9800
C2—C3	1.432 (2)	C14—H14C	0.9800
C3—C4	1.491 (2)	C15—H15A	0.9800
C4—H4A	0.9800	C15—H15B	0.9800
C4—H4B	0.9800	C15—H15C	0.9800
C4—H4C	0.9800		
			
O2^i^—Ni1—O2	180.0	O2—C5—C6	116.45 (15)
O2^i^—Ni1—O1^i^	90.81 (5)	C2—C5—C6	120.89 (15)
O2—Ni1—O1^i^	89.19 (5)	C5—C6—H6A	109.5
O2^i^—Ni1—O1	89.19 (5)	C5—C6—H6B	109.5
O2—Ni1—O1	90.81 (5)	H6A—C6—H6B	109.5
O1^i^—Ni1—O1	180.0	C5—C6—H6C	109.5
O2^i^—Ni1—O3^i^	90.17 (5)	H6A—C6—H6C	109.5
O2—Ni1—O3^i^	89.84 (5)	H6B—C6—H6C	109.5
O1^i^—Ni1—O3^i^	88.50 (5)	C12—C7—C8	119.80 (16)
O1—Ni1—O3^i^	91.50 (5)	C12—C7—N1	118.86 (15)
O2^i^—Ni1—O3	89.83 (5)	C8—C7—N1	121.32 (15)
O2—Ni1—O3	90.16 (5)	C9—C8—C7	119.33 (16)
O1^i^—Ni1—O3	91.50 (5)	C9—C8—H8	120.3
O1—Ni1—O3	88.50 (5)	C7—C8—H8	120.3
O3^i^—Ni1—O3	179.999 (1)	C8—C9—C10	121.12 (17)
C1—O1—Ni1	118.40 (11)	C8—C9—H9	119.4
C5—O2—Ni1	127.18 (11)	C10—C9—H9	119.4
C13—O3—Ni1	121.20 (11)	C11—C10—C9	118.91 (17)
C1—N1—N2	112.16 (14)	C11—C10—H10	120.5
C1—N1—C7	130.19 (14)	C9—C10—H10	120.5
N2—N1—C7	117.63 (14)	C10—C11—C12	120.87 (17)
C3—N2—N1	105.38 (14)	C10—C11—H11	119.6
C13—N3—C14	120.62 (15)	C12—C11—H11	119.6
C13—N3—C15	122.03 (15)	C11—C12—C7	119.92 (17)
C14—N3—C15	117.34 (14)	C11—C12—H12	120.0
O1—C1—N1	123.46 (15)	C7—C12—H12	120.0
O1—C1—C2	131.43 (16)	O3—C13—N3	123.95 (16)
N1—C1—C2	105.10 (14)	O3—C13—H13	118.0
C5—C2—C1	123.45 (16)	N3—C13—H13	118.0
C5—C2—C3	131.18 (16)	N3—C14—H14A	109.5
C1—C2—C3	105.17 (14)	N3—C14—H14B	109.5
N2—C3—C2	112.18 (15)	H14A—C14—H14B	109.5
N2—C3—C4	118.10 (16)	N3—C14—H14C	109.5
C2—C3—C4	129.68 (16)	H14A—C14—H14C	109.5
C3—C4—H4A	109.5	H14B—C14—H14C	109.5
C3—C4—H4B	109.5	N3—C15—H15A	109.5
H4A—C4—H4B	109.5	N3—C15—H15B	109.5
C3—C4—H4C	109.5	H15A—C15—H15B	109.5
H4A—C4—H4C	109.5	N3—C15—H15C	109.5
H4B—C4—H4C	109.5	H15A—C15—H15C	109.5
O2—C5—C2	122.64 (15)	H15B—C15—H15C	109.5
			
O2^i^—Ni1—O1—C1	−156.12 (12)	N1—N2—C3—C2	0.82 (19)
O2—Ni1—O1—C1	23.88 (12)	N1—N2—C3—C4	178.73 (15)
O1^i^—Ni1—O1—C1	20 (22)	C5—C2—C3—N2	−175.27 (17)
O3^i^—Ni1—O1—C1	113.74 (12)	C1—C2—C3—N2	−0.32 (19)
O3—Ni1—O1—C1	−66.26 (12)	C5—C2—C3—C4	7.1 (3)
O2^i^—Ni1—O2—C5	97 (5)	C1—C2—C3—C4	−177.93 (17)
O1^i^—Ni1—O2—C5	157.07 (13)	Ni1—O2—C5—C2	10.4 (2)
O1—Ni1—O2—C5	−22.93 (13)	Ni1—O2—C5—C6	−171.37 (10)
O3^i^—Ni1—O2—C5	−114.44 (13)	C1—C2—C5—O2	8.4 (3)
O3—Ni1—O2—C5	65.57 (13)	C3—C2—C5—O2	−177.46 (16)
O2^i^—Ni1—O3—C13	−39.74 (12)	C1—C2—C5—C6	−169.79 (15)
O2—Ni1—O3—C13	140.26 (12)	C3—C2—C5—C6	4.4 (3)
O1^i^—Ni1—O3—C13	51.07 (13)	C1—N1—C7—C12	−154.26 (17)
O1—Ni1—O3—C13	−128.93 (13)	N2—N1—C7—C12	23.8 (2)
O3^i^—Ni1—O3—C13	82 (18)	C1—N1—C7—C8	26.9 (3)
C1—N1—N2—C3	−1.06 (18)	N2—N1—C7—C8	−155.00 (15)
C7—N1—N2—C3	−179.46 (14)	C12—C7—C8—C9	−2.4 (2)
Ni1—O1—C1—N1	164.19 (12)	N1—C7—C8—C9	176.44 (15)
Ni1—O1—C1—C2	−16.5 (2)	C7—C8—C9—C10	0.7 (2)
N2—N1—C1—O1	−179.70 (14)	C8—C9—C10—C11	1.3 (3)
C7—N1—C1—O1	−1.6 (3)	C9—C10—C11—C12	−1.6 (3)
N2—N1—C1—C2	0.86 (18)	C10—C11—C12—C7	0.0 (3)
C7—N1—C1—C2	179.00 (16)	C8—C7—C12—C11	2.0 (2)
O1—C1—C2—C5	−4.3 (3)	N1—C7—C12—C11	−176.81 (15)
N1—C1—C2—C5	175.11 (15)	Ni1—O3—C13—N3	172.39 (12)
O1—C1—C2—C3	−179.71 (17)	C14—N3—C13—O3	0.4 (3)
N1—C1—C2—C3	−0.33 (17)	C15—N3—C13—O3	179.29 (16)

Symmetry codes: (i) −*x*+1, −*y*+2, −*z*+1.
